# Using a novel methodology to map Post-COVID services for children and young people in England: a web-based systematic search

**DOI:** 10.1186/s12913-024-11283-7

**Published:** 2024-07-29

**Authors:** Fiona Newlands, Lana Fox-Smith, Sonia Balakrishnan, Gareth Lord, Trudie Chalder, Emma Dalrymple, Tamsin Ford, Anthony Harnden, Isobel Heyman, Shamez N Ladhani, Snehal M Pinto Pereira, Terry Y Segal, Terence Stephenson, Elizabeth Whittaker, Roz Shafran

**Affiliations:** 1 UCL Great Ormond Street Institute of Child Health, 30 Guilford Street, London, WC1N 1EH England, UK; 2 King’s College London, De’Crespigny Park, London, SE5 8AF England, UK; 3 NHS England, Quarry House, Leeds, LS2 7PD England, UK; 4 Institute of Psychiatry, Psychology and Neuroscience, King’s College London, De’Crespigny Park, London, SE5 8AF England, UK; 5 Department of Psychiatry, University of Cambridge, Cambridge, England, UK; 6 Nuffield Department of Primary Care Health Sciences, University of Oxford, Oxford, England, UK; 7 Paediatric Infectious Diseases Research Group, St George’s University of London, London, England, UK; 8 Division of Surgery & Interventional Science, Faculty of Medical Sciences, University College London, London, WC1E 6BT England, UK; 9 Univerisity College London Hospital, 235 Euston Rd, London, NW1 2BU England, UK; 10 Imperial College Healthcare NHS Trust, The Bays, S Wharf Rd, London, W2 1NY England, UK

**Keywords:** Post-COVID services, Long COVID, Children and young people, Paediatric, SARS-CoV-2

## Abstract

**Background:**

Post-COVID Condition (PCC), also known as ‘Long COVID,’ refers to persistent symptoms following a coronavirus 2 (SARS-CoV-2) infection. The prevalence of PCC in children and adolescents varies, impacting multiple body systems and affecting daily functioning. Specialised paediatric hubs were established in England to address the needs of young individuals with PCC. Additional local services also emerged, yet patients report challenges accessing services. To better understand the landscape of paediatric PCC services, we used a novel methodology using a web-based systematic search.

**Methods:**

A web-based search was conducted in July 2023 using DEVONagent Pro. Search terms related to Long COVID and Pediatrics in England. Eligible sources providing information on PCC services for children and young people were included. A supplementary manual search and NHS England Post-COVID Network were also consulted. Data extraction included service location, characteristics, and referral pathways. Population estimates were derived from UK Census data.

**Results:**

Among 342 identified records, 27 services met eligibility criteria, distributed unevenly across regions. Specialised hubs covered 13 locations, while additional services were concentrated in the South of England and London. Services varied in team composition, age range treated, and support offered. A lack of standardised approaches for paediatric PCC was evident.

**Discussion:**

We used a novel methodology for systematically mapping online resources, providing valuable insights into service accessibility and aiding the identification of potential gaps. We observed geographical disparities in access to paediatric PCC services and the absence of standardised approaches in managing symptoms. Given the challenges faced by young individuals seeking support for their PCC the need for equitable and standardised care became apparent. The study contributes to closing the research-practice gap and calls for further research to identify effective treatments for paediatric PCC, acknowledging the diversity of reported symptoms and the importance of tailored approaches.

**Supplementary Information:**

The online version contains supplementary material available at 10.1186/s12913-024-11283-7.

## Introduction

Post-COVID Condition (PCC), syndrome or ‘Long COVID’ are terms used to describe enduring symptoms experienced after a coronavirus 2 (SARS-CoV-2) infection. The World Health Organization (WHO) defines the condition in children and adolescents as occurring “in individuals with a history of confirmed or probable SARS-CoV-2 infection when experiencing symptoms lasting at least 2 months which initially occurred within 3 months of acute COVID-19”. Prevalence estimates in children and young people (CYP) vary greatly, ranging from 1 to 70% [1–3]; however, a recent study indicates a more conservative estimate of 7% of young people consistently fulfilling the WHO definition over a 24 month period [4]. Over 200 symptoms have been associated with PCC impacting different systems in the body including the respiratory, cardiovascular, neuropsychological, digestive, circulatory, musculoskeletal, and genitourinary systems [5–7]. These symptoms have an impact on everyday functioning and may fluctuate over time [8].

In July 2020, ‘Your COVID Recovery’ was launched by NHS England with the goal of providing practical advice and guidance for individuals recovering from COVID-19 [9]. However, recognising the need for more extensive support, in June 2021, NHS England established specialised PCC hubs with Multidisciplinary Teams (MDTs) to aid CYP living with the condition. A dedicated £100 million in funding was allocated to set up 15 specialist hubs for CYP (https://www.england.nhs.uk/2021/06/nhs-sets-up-specialist-young-peoples-services-in-100-million-long-covid-care-expansion). By April 2023, 13 of these specialised paediatric hubs were operational. Initially focusing on supported self-management post-assessment, the hubs later shifted to providing more intensive support, acknowledging the increased need for input. The level of support offered at these hubs varies, but the universal objective is to provide comprehensive multidisciplinary assessment and management support. This includes physical, cognitive, and mental health assessments, along with diagnostic tests and management strategies, including referrals to other specialist services as needed. (https://www.england.nhs.uk/coronavirus/post-covid-syndrome-long-covid/).

In addition to these specialised hubs, England is served by 229 NHS Trusts [10], the majority of which operate at least one general paediatric service, with larger Trusts encompassing multiple hospitals spread across a broad geographical area. These general paediatric services typically act as the primary point of contact for CYP seeking support for their PCC symptoms following an initial consultation with a general practitioner (GP) and preceding any referral to one of the 13 specialised paediatric Post-COVID hubs.

Despite the presence of these general paediatric services and the establishment of specialist hubs, patients describe the process of accessing services as complex, difficult and exhausting [11, 12]. Patients report that GPs are not always aware of rehabilitation services, feel a lack of clarity in the defined pathways, and express having to independently research their own routes to access the necessary support [12]. Similarly, a recent study on CYP reported challenges trying to access services because of their locations and availability of appointments along with difficulties associated with a lack of understanding about the services and referral routes in place to help CYP with PCC [13].

Considering these challenges, there is a need to better identify the services available, where they are located, the treatments they offer and the referral pathways into them. However, this is not an easy task and a new methodology is required with clear parameters for inclusion and exclusion, eliciting material from the internet rather than exclusively from traditional academic sources. We propose to adapt established procedures for conducting systematic reviews of research literature for the novel purpose of collecting and synthesising information from online sources. Such a “web-based systematic search” can be used to comprehensively map paediatric Post-COVID services across England. This innovative methodology recognises the role of online resources in disseminating information and facilitating access to services and treatment for those affected by paediatric PCC, and would provide a picture of the geographical spread, and type of services available for CYP seeking support for their PCC symptoms.

Therefore, the aim of the study was to identify and map paediatric Post-COVID provision across England with the specific objectives to: (1) examine the prevalence of specialist services for CYP with PCC, their geographical distribution and service characteristics such as referral pathways, and treatment age range; and (2) trial a novel methodology to identify and describe these services.

## Methods

This systematic web-based search was performed and reported according to items 1–10 of the PRISMA guidelines [14].

### Eligibility

Websites or resources (e.g., PDFs) including details of services for CYP with symptoms of PCC in England were included. Websites were excluded if they did not contain information on paediatric Post-COVID provision in England or only provided support for adults (age 18 +). We also excluded any results for individual clinicians not providing support as part of a service.

### Search strategy

DEVONagent Pro (https://www.devontechnologies.com/apps/devonagent) is a software package that searches multiple online sources and provides a summary of results based on specific search terms. The software was used to conduct a web-based search on 25th July 2023 Search terms related to four concepts: (1) Post-COVID Condition; (2) Children and young people; (3) Clinical services; and (4) England. Search terms were developed in collaboration with a librarian. See Additional file 2 for the search settings and a full list of search terms used.

A further manual search was conducted using Safari to capture any services not picked up by DEVONagent Pro using a combination of the search terms above. Services were also identified via the NHS England Post-COVID Network.

The data on the number of CYP served by the service catchment area and residing in each region of England were obtained by extracting information from population estimates sourced from the UK Census data of 2022 [15].

### Site selection and data extraction

Results from the DEVONagent PRO search were exported to an excel spreadsheet. Results identified in the manual search were added to the spreadsheet along with those found via the NHS England Post-COVID working group. Results were screened independently by SB and LFS with any queries verified by a second reviewer (FN or RS). Searches were marked as ‘included’, ‘excluded’ or ‘requires more information’ if it was unclear whether it should be included e.g., in some cases it was unclear whether a service provided treatment for children and young people. Any services marked ‘requires more information’ were contacted via telephone and/or email. Services that responded were included/ excluded in line with the eligibility criteria.

Data relating to the location of the service and details describing the service, including the type of service, the age range seen, the type of organisation (NHS or private), the available specialities within the service and referral pathways were extracted. A proportional symbol map was created using the software Flourish (https://flourish.studio/visualisations/maps/) as a visual representation of the spread of services across England.

## Results

**Fig. 1:**
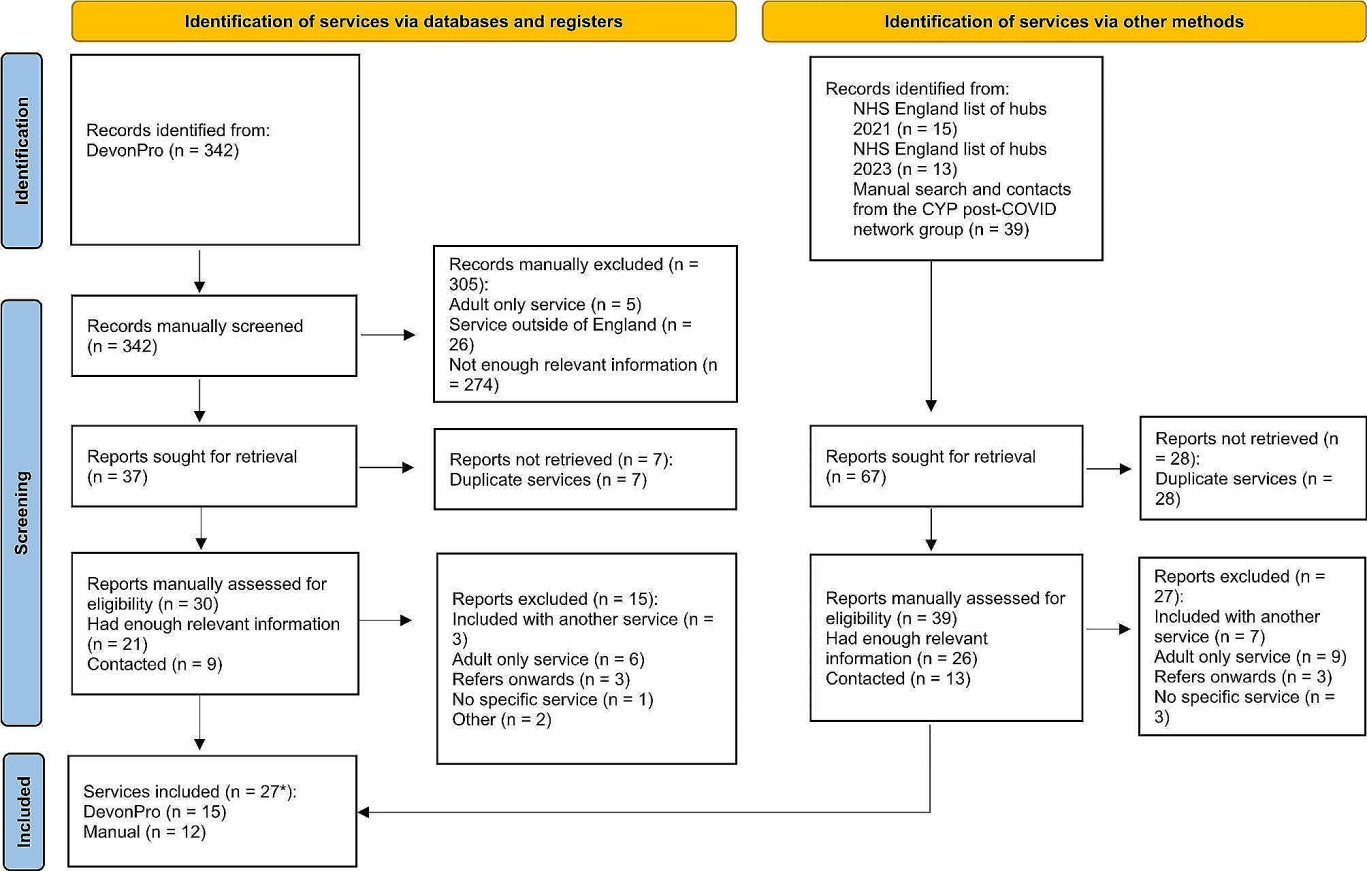
PRISMA diagram of paediatric Post-COVID services across England. *Hull and East Riding are a joint service and have been listed as one under Hull University Teaching Hospitals NHS Trust. University Hospitals of Leicester NHS Trust covers NHS Leicester and Leicestershire and Rutland. South East London Service includes Guy’s and St Thomas and Kings College Hospital. Cambridge University Hospitals NHS Foundation Trust includes Addenbrooke’s Hospital. University College London Hospitals NHS Foundation Trust and Imperial College London are part of the Pan-London service and have been grouped as UCLH. *From*: Page MJ, McKenzie JE, Bossuyt PM, Boutron I, Hoffmann TC, Mulrow CD, et al. The PRISMA 2020 statement: an updated guideline for reporting systematic reviews. BMJ 2021;372:n71. doi: 10.1136/bmj.n71. For more information, visit: [16]

**Fig. 2:**
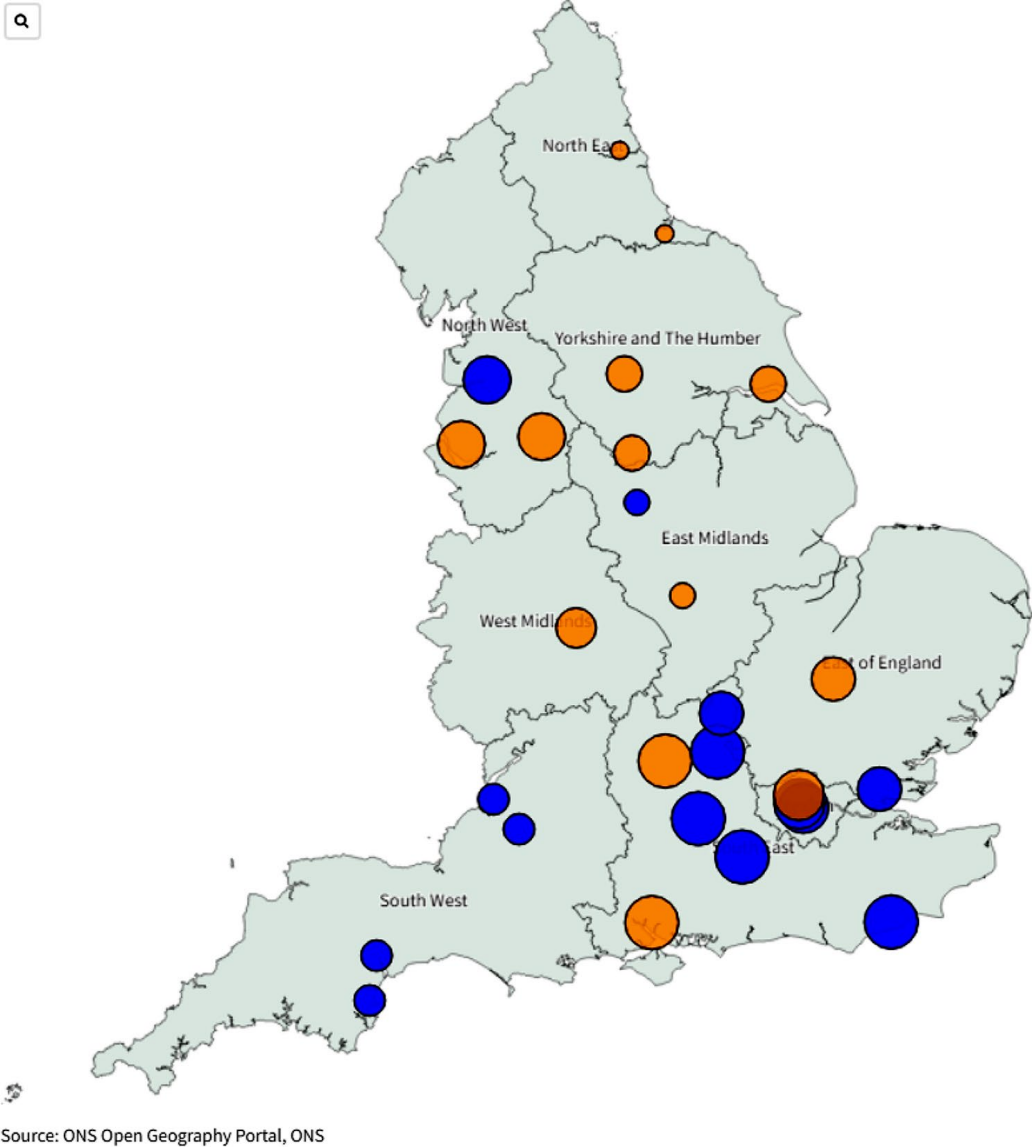
Paediatric Post-COVID Services across England. Orange dots represent the 13 specialist Paediatric Post-COVID hubs set up by NHS-E. Blue dots represent other services in England that offer services for CYP with PCC. The size of the dots are determined by the 2021 census data [15], reflecting the number of CYP (0–18 years old) within each region of England

342 records were identified by DEVONagent Pro search. After removing duplicates (*n* = 7) and excluding those results that did not meet the eligibility criteria or there was not enough relevant information, 27 services were included in the search (15 DEVONagent and 12 via other methods). See Fig. 1. Services were spread across the South East (*n* = 6), the South West (*n* = 4), the East of England (*n* = 3), Yorkshire and the Humber (*n* = 3), North West (*n* = 3) London (*n* = 3), East Midlands (*n* = 2) the North East (*n* = 2) and the West Midlands (*n* = 1). See Fig. 2 (Table 1).

**Table 1:** List of Paediatric Post-COVID services in England

Region	Service Number	Service	Specialist NHS Paediatric Post-COVID hubs	Age range*	Referral pathway	Additional information	# CYP (0–18) in region from 2021 census data [15]
North East	i	South Tees Hospitals NHS Foundation Trust	Y	NR	Through GP or secondary care referrals		561,409
	ii	The Newcastle Upon Tyne Hospitals NHS Foundation Trust	Y	NR	NR	Listed as one of the NHS paediatric hubs but no specific website of information	
North West	iii	Alder Hey Children’s NHS Foundation Trust	Y	0–18	GP referral		1,669,862
	iv	Manchester University NHS Foundation Trust	N	0–18	GP referral		
	v	Lancashire and South Cumbria NHS Foundation Trust	N	under 18’s	GP referral		
Yorkshire and the Humber	vi	Sheffield Children’s NHS Foundation Trust	Y	NR	NR	Listed as one of the NHS paediatric hubs but no specific website of information	1,222,859
	vii	Leeds Teaching Hospitals NHS Trust	Y	< 16yrs old	Secondary care referral (paediatric or GP)		
	viii	Hull University Teaching Hospitals NHS Trust	Y	NR	Through hospital/community based paediatricians referrals	Includes Hull and East Riding service	
East Midlands	ix	University Hospitals of Leicester NHS Trust	Y	< 16yrs old	GP referral	Includes NHS Leicester, Leicestershire and Rutland	1,060,853
	x	Joined Up Care Derbyshire	N	Prioritises under 16’s, but a child and adult service	GP referral		
West Midlands	xi	Birmingham Women’s and Children’s NHS Foundation Trust	Y	NR	Referral via community child-development service, community therapies, CAMHS, primary care.		1,382,103
East of England	xii	Cambridge University Hospitals NHS Foundation Trust	Y	0–18	NR	Includes Addenbrookes	1,419,121
	xiii	Mid and South Essex NHS foundation trust	N	child and adult service	Through GP/healthcare professional referrals		
	xiv	Bedfordshire, Luton & Milton Keynes Hospital	N	any age	GP referral		
London	xv	University College London Hospitals NHS Foundation Trust	Y	0–18	Paediatrician/GP referrals + referral contacts for healthcare staff	Part of the Pan London Service jointly run with Evelina Children’s Hospital	1,998,880
	xvi	The London Clinic*	N	adults and children	Self-referral. Book appointment to see GP. Also, Existing referrals from GP	Private hospital in London	
	xvii	Guy’s and St Thomas’ NHS Foundation Trust	N	NR	GP referral or internal e.g. respiratory clinics	Part of South East London Long COVID service - clinics at Kings College Hospital and Guy’s and St Thomas’	
South East	xviii	Oxford University Hospitals NHS Foundation Trust	Y	NR	NR	Listed as one of the NHS paediatric hubs but no specific website of information	2,070,084
	xix	Solent NHS Trust	Y	under 18’s	GP referral	Covers 6 services: Portsmouth Long COVID Service, South East Hampshire Long COVID Service, Southampton Long COVID Service, South West Hampshire Long COVID Service, North & Mid Hampshire Long COVID Service, Isle of Wight Long COVID Service	
	xx	East Sussex Healthcare NHS Trust	N	adult and child services (under 18’s)	Primary care referral		
	xxi	Royal Surrey NHS Foundation Trust	N	under 18’s and adult service	internal and external referral (GP)		
	xxii	Buckinghamshire Healthcare NHS Trust	N	under 16’s	GP referral		
	xxiii	Royal Berkshire NHS Foundation Trust	N	children and adults	Through GP referrals + patient questionnaire		
South West	xxiv	Royal United Hospitals Bath	N	under 18’s	all referrals to us must be made by a hospital consultant, GP or other health professional.		1,159,174
	xxv	Bristol Royal Hospital for Children/ South West Long Covid Hub	Y	NR	GP referral	As of 27th February 2023 this service no longer accepting new referrals.	
	xxvi	Royal Devon and Exeter NHS Foundation Trust	N	under 18’s	GP referral	North and East Devon Formulary	
	xxvii	South and West Devon Formulary and Referral NHS	N	under 16’s	GP referral		

NR = not reported. * NR indicates that the service is for paediatric patients, but there was no available information on the particular age range of children treated within the service

Thirteen of the 15 specialist PCC hub services initially given funding by NHS England were in operation; Queen Alexandra and University Hospital Southampton NHS Foundation Trust were not operating and Bristol Royal Hospital for Children/ South West Long Covid Hub had stopped receiving PCC referrals as of 27th February 2023. There was no webpage or information available for three of the 13 hubs listed as operating in April 2023 (ii; vi; xviii). 26 of the services were in the public sector and one of the services was in the private sector (xvi).

Services included a range of health care professionals including doctors or paediatricians, nurses (including clinical nurse specialists and nurse consultants), physiotherapists, psychological practitioners (including psychiatrists, senior clinical psychologists, assistant psychologists, psychotherapists, psychological practitioners) occupational therapists, speech and language therapists, dieticians and rehab support workers. Four services referenced their MDT (viii; xi; xv; xxi) with one including specialist roles in adolescent medicine, respiratory, infectious diseases, rheumatology, cardiology, neurology, psychiatry (xxi). Two services included primary care professionals to assess PCC (xxvi; xxvii) and one stated the service consisted of general practitioners (GPs - also referred to as Family Physicians in some countries) with a specialist interest in PCC and a hospital consultant (v).

The type of support offered at each service included holistic or multi-disciplinary assessment including physical, physiotherapy and psychological assessments and occupational therapy, PCC assessments, mental health support and fatigue services, self-care and self-management resources, support groups, advice and education. Three services also indicated that they would refer on to other specialist services e.g. Ear, Nose and Throat (ENT).

Eight services specified they saw young people aged 0–18 years old (iii; iv; v; xii; xv; xix; xxiv; xxvi). Six offered services to both children and adults (xiii; xiv; xvi; xx; xxi; xxiii). Eight services did not provide specific details regarding the age range of the treated population. These services were identified either as one of the 13 specialist paediatric PCS or made a general reference to treating children without explicitly specifying the age groups (i; ii; vi; viii; xi; xvii; xviii; xxv). Four saw under 16s (vii; ix; xxii; xxvii) and one was a child and adult service but prioritised under 16’s (x).

Only one service took self-referrals (xvi- Private healthcare service). The majority of services (*n* = 21) stated that referral pathways were via a GP, paediatrician, hospital consultant or secondary care/ health care professional (i; iii; iv; v; vii; ix; x; xiii; xiv; xv; xvi; xvii; xix; xx; xxi; xxii; xxiii; xxiv; xxv; xxvi; xxvii). One service offered hospital/community-based paediatricians referrals (viii). Another service mentioned referrals via community child-development service, community therapies, CAMHS, or primary care (xi).

Websites for two services included details on the number of referrals they had received. Bedfordshire, Luton & Milton Keynes Hospital estimated 20–30 people were referred per week, although it was not stated how many of these were CYP. Royal Devon and Exeter NHS Foundation Trust stated that as of 19/08/22, there had been 323 initial appointments with CYP and 293 follow ups.

## Discussion

This study aimed to use a novel methodology to identify specialist services for CYP with PCC including their geographical distribution and service characteristics such as referral pathways and age range treated. Our search revealed 27 PCC services providing support for CYP. While the 13 specialist Paediatric PCC hubs were more evenly distributed across England, the majority of other services were located in the South of England and London. This is reflective of the significant geographical variation in access to services across the country highlighted by the Commissioning Guidance for Post-COVID services [17]. The challenges encountered in identifying services for PCC are likely applicable to other conditions and highlight a broader issue of transparency within services. Greater clarity and accessibility to information about available services are crucial for ensuring equitable access to specialised care.

Information on referral pathways, the age of young people seen in the clinic and the roles within a team were vague and not consistently available, reflecting some of the challenges faced by patients and families trying to access support [11, 12]. Findings suggest an absence of a standardised approach to managing symptoms, which is unsurprising given the lack of information on best practice for the treatment of paediatric PCC.

In terms of treatment, the current evidence-based for paediatric PCC is limited and given the multitude of symptoms associated with the condition [2, 6, 7], it’s unlikely that one treatment option would be sufficient. In the absence of a ‘magic bullet’ for treating PCC, the management has involved: excluding other underlying medical conditions; investigating for other causes of fatigue such as obstructive sleep apnoea; and focussing on hydration, regular meals and salt intake, exercise, sleep hygiene, breathing exercises for dysfunctional breathing patterns, analgesia, and fludricortisone or beta-blockers for postural orthostatic tachycardia [18]. However, the lack of evidence demonstrating the effectiveness of highlights the need for further research to guide healthcare providers and policymakers in developing a standardised management approach that can be tailored to address the wide range of symptoms experienced by patients with PCC. There is a compelling case to explore the possibility of these services collaborating to form a unified platform for evaluating interventions. This collaborative effort could significantly enhance the evaluation process and ultimately improve patient care.

Four services stated they saw CYP only up to 16 years old, creating a gap in provision for those transitioning from child to adult services. This gap is particularly alarming given that older children are at a greater risk of experiencing enduring symptoms [7] Additionally, this is a departure from commissioning guidance, which suggests services should offer support to 18-year-olds still in school [17]. It’s important to consider the immense impact on life opportunities when individuals are not functioning during this adolescence including repercussions on education and life choices.

A secondary aim of this study was to trial a novel approach to searching for relevant resources by conducting a web-based systematic search to identify these Paediatric PCC services. This approach was trialled in acknowledgement of the important role of online resources in disseminating information and facilitating access to treatment and services for CYP affected by PCC. The novelty of the approach lies in two key aspects. First, the application of the adoption of systematic search processes previously only used in the academic literature to online resources. Such a strategy to service identification ensures a rigorous approach. Second, the tool’s ability to produce search results with high sensitivity and specificity. Importantly, this methodology can be adapted and applied to identify other specialised healthcare services.

While this methodology was a helpful component of the identification of the services, it was not entirely comprehensive, in part due to the lack of publicly available information. As a result, a further manual search was required which identified additional services (*n* = 12). This indicates that while available software packages may help streamline searches, an additional manual search helps to provide a more comprehensive and exhaustive compilation of relevant services, particularly in contexts where specific, localised resources might not be easily retrieved.

### Practical implications

By collating and consolidating information from online sources, this search aids the understanding of the accessibility of services available to children and young people seeking support for their PCC symptoms. The novel methodology provides a viable alternative to traditional research reviews and can help close the research-practice gap. However, a manual search is also required for a comprehensive overview of relevant sources. It is important to note that this list is not exhaustive, and there may be other services, including general paediatrics and private services, that provide treatment for CYP with PCC but do not have an online presence. Identifying referral pathways and treatment options for affected individuals was complex and suggests the need for a platform to help patients locate local services. For example, NHS talking therapies postcode finder into online resources can serve as a model for streamlining access to local services. Public use of these data must allow for evolution and updating, for example, the addition of those centres not initially identified through this methodology to be added to the listings of available centres.

### Strengths and limitations

The study addresses a knowledge gap in understanding the services available for CYP with PCC. This utilisation of the DEVONagent Pro software package proved to be highly effective in filtering through vast amounts of information to provide relevant and concise search outcomes. Such functionality is particularly valuable to reviewers and information specialists who require efficient and comprehensive search tools. The subsequent manual search further supplemented the gathered data, ensuring a more exhaustive compilation of available services.

The reliance on online sources might have inadvertently excluded services not easily accessible or represented online, potentially leading to an incomplete representation of available resources. Additionally, as we understand more about the nature of PCC, our understanding of the numbers and types of services required to meet the needs of individuals will evolve. The variations in the terminology used across different sources might have led to missed or underrepresented services, highlighting the need for standardisation and consistency in reporting and categorising PCC-related services. The DEVONagent Pro search was limited to a maximum of 1000 results per plugin to ensure the results remained manageable and relevant. While this limit may exclude some potentially useful data, it strikes a balance between comprehensiveness and practicality, allowing for more efficient analysis and review. Finally, the software package is currently only available for use on Apple products, which limits the accessibility for users on other platforms and may reduce its adoption more widely.

## Conclusion

This study presents a novel methodology for collating and synthesising online services/resources in a systematic way. It highlights some of the challenges experienced by CYP and their families trying to access services for their PCC symptoms. There is a need for more equitable care for young people across England. Online guidance for families seeking information on treatments and symptom management is required. Further studies are required to identify effective treatments for children and young people living with PCC. Given the number of diverse symptoms that CYP report it is likely that a treatment approach which focuses on coping strategies may lead to tailored approaches. Simultaneously, research investigating mechanisms of symptom persistence and mechanism of change in treatment trials, alongside research into the best service models and referral pathways, would be beneficial. These efforts are crucial for advancing our understanding of PCC and improving care outcomes for CYP.

### Electronic supplementary material

Below is the link to the electronic supplementary material.

Supplementary Material 1

## Data Availability

All legitimate requests should be made in writing to the corresponding author.
