# Supplementary material for: Using a novel methodology to map Post-COVID services for children and young people in England: a web-based systematic search
Source: BMC Health Serv Res. 2024 Jul 29;24:863. doi: 10.1186/s12913-024-11283-7 (PMC11288108; doi:10.1186/s12913-024-11283-7)
Supplement: Supplementary file 1 — Supplementary Material 1 [file 12913_2024_11283_MOESM1_ESM.docx]

**Additional file 1**

Search set settings:

Language: English; “Ignore Diactrics” checked; “Fuzzy” checked (allow alternative spelling); Similar pages NOT filtered; Archived pages NOT filtered. Search terms in Title, Text, URL, Keywords, Description; HTML & XHTML pages, Atom, RSS & JSON feeds, Plain text documents, PDFs, and Microsoft word documents will be searched; Searches across Plugins: Bing, Google, Yahoo!; Maximum results limit per Plugin: 1000.

Search terms:

The ‘default query’ or primary set of search terms were: “Long COVID*” OR “Post COVID*” OR “postCOVID” OR “post acute COVID” OR “chronic COVID” OR “long term COVID” OR “long haul COVID” OR “post acute sequelae OR “Post COVID Syndrome*” OR PCS. The ‘secondary query’ used as parameters to refine the search results were: Paediatri* OR Pediatri* OR Child* OR "Young Person*" OR "Young People*" OR "Young Adult*" OR Teen* OR Youth* OR adolescen* AND England* OR UK* OR "Great Britain*" OR "United Kingdom*" AND Service* OR Clinic* OR Hub* OR Treat* OR Interven* OR Manag* OR Rehab* OR Assistance* OR practice* OR care OR outpatient* OR “out-patient*” OR unit* or center* or centre* AND NHS OR “National Health Service”.
